# Improving Endodontic Radiograph Interpretation with TV-CLAHE for Enhanced Root Canal Detection

**DOI:** 10.3390/jcm14155554

**Published:** 2025-08-06

**Authors:** Barbara Obuchowicz, Joanna Zarzecka, Michał Strzelecki, Marzena Jakubowska, Rafał Obuchowicz, Adam Piórkowski, Elżbieta Zarzecka-Francica, Julia Lasek

**Affiliations:** 1 Department of Conservative Dentistry with Endodontics, Jagiellonian University Collegium Medicum, Montelupich 4, 31-155 Cracow, Poland; barbara.obuchowicz@uj.edu.pl (B.O.); j.zarzecka@uj.edu.pl (J.Z.); mjakubowska@uks.com.pl (M.J.); 2 Institute of Electronics, Lodz University of Technology, 93-590 Lodz, Poland; 3 Department of Diagnostic Imaging, Jagiellonian University Medical College, 30-663 Krakow, Poland; r.obuchowicz@gmail.com; 4 Department of Biocybernetics and Biomedical Engineering, AGH University of Krakow, 30-059 Krakow, Poland; 5 Department of Prosthodonics with Orthodontics, Institute of Dentistry Jagiellonian University Medical College, 30-059 Krakow, Poland; elzbieta.zarzecka@gmail.com; 6 Faculty of Geology, Geophysics and Environmental Protection, AGH University of Krakow, 30-059 Krakow, Poland

**Keywords:** root canal anatomy, image enhancement, CLAHE, dental radiography, endodontics

## Abstract

**Objective:** The accurate visualization of root canal systems on periapical radiographs is critical for successful endodontic treatment. This study aimed to evaluate and compare the effectiveness of several image enhancement algorithms—including a novel Total Variation–Contrast-Limited Adaptive Histogram Equalization (TV-CLAHE) technique—in improving the detectability of root canal configurations in mandibular incisors, using cone-beam computed tomography (CBCT) as the gold standard. A null hypothesis was tested, assuming that enhancement methods would not significantly improve root canal detection compared to original radiographs. **Method:** A retrospective analysis was conducted on 60 periapical radiographs of mandibular incisors, resulting in 420 images after applying seven enhancement techniques: Histogram Equalization (HE), Contrast-Limited Adaptive Histogram Equalization (CLAHE), CLAHE optimized with Pelican Optimization Algorithm (CLAHE-POA), Global CLAHE (G-CLAHE), k-Caputo Fractional Differential Operator (KCFDO), and the proposed TV-CLAHE. Four experienced observers (two radiologists and two dentists) independently assessed root canal visibility. Subjective evaluation was performed using an own scale inspired by a 5-point Likert scale, and the detection accuracy was compared to the CBCT findings. Quantitative metrics including Peak Signal-to-Noise Ratio (PSNR), Signal-to-Noise Ratio (SNR), image entropy, and Structural Similarity Index Measure (SSIM) were calculated to objectively assess image quality. **Results:** Root canal detection accuracy improved across all enhancement methods, with the proposed TV-CLAHE algorithm achieving the highest performance (93–98% accuracy), closely approaching CBCT-level visualization. G-CLAHE also showed substantial improvement (up to 92%). Statistical analysis confirmed significant inter-method differences (*p* < 0.001). TV-CLAHE outperformed all other techniques in subjective quality ratings and yielded superior SNR and entropy values. **Conclusions:** Advanced image enhancement methods, particularly TV-CLAHE, significantly improve root canal visibility in 2D radiographs and offer a practical, low-cost alternative to CBCT in routine dental diagnostics. These findings support the integration of optimized contrast enhancement techniques into endodontic imaging workflows to reduce the risk of missed canals and improve treatment outcomes.

## 1. Introduction

The accurate identification of root canals is a cornerstone of successful endodontic treatment. Missed or undetected canals are a well-established cause of persistent apical periodontitis, endodontic failure, and the need for retreatment or surgical intervention [1]. Studies have consistently demonstrated that missed canals significantly increase the risk of treatment failure, with some reports indicating failure rates exceeding 80% when canals are left untreated, compared to approximately 60% when all canals are identified and properly filled [2,3]. This is particularly true in anatomically complex teeth, such as maxillary first molars with second mesiobuccal (MB2) canals or mandibular molars with C-shaped canals—configurations that are often underdiagnosed due to their morphological intricacy [4,5]. The small size of the relevant anatomical structures often makes it difficult to capture a clear anatomical outline. Additionally, the complexity of the region is heightened by overlapping structures that contribute to “anatomical noise” [6].

Conventional periapical radiographs remain the most commonly used imaging modality in routine dental practice due to their low cost, accessibility, and relatively low radiation dose [7,8]. However, their two-dimensional nature poses limitations in detecting superimposed or narrow canals, particularly in teeth with complex internal anatomy or pulp canal calcification [9]. In these cases, the likelihood of missing a canal increases, potentially leading to chronic infection and post-treatment disease. The advent of cone-beam computed tomography (CBCT) has significantly enhanced clinicians’ ability to visualize root canal systems in three dimensions [10]. Yet, due to cost, availability, and radiation concerns, CBCT is not always feasible for every patient or clinical setting [11,12], enhancing the diagnostic value of standard periapical radiographs through digital image processing techniques has become a critical area of research.

Accurate classification of root canal morphology in mandibular incisors remains clinically challenging due to anatomical variability and radiographic limitations. Vertucci’s seminal study on extracted teeth showed that 70–75% had a Type I configuration (1-1), while 25–30% exhibited more complex types (II–IV), revealing a significant subset with hidden or merging canals [13,14,15,16].

To address the inherent limitations of 2D imaging, various image enhancement and diagnostic technologies have been developed. Magnification tools, such as dental operating microscopes (DOMs), and ultrasonic instrumentation have been proven to increase the detection rate of challenging canal morphologies like MB2 or calcified canals [17]. In parallel, artificial intelligence (AI)-driven diagnostic models are emerging as potential decision-support tools capable of analyzing dental radiographs and CBCT data to identify anatomical landmarks and hidden canals with a high degree of precision.

Given the profound clinical impact of undetected canals, there is a need to optimize detection strategies within the context of routine diagnostic radiography. This work focuses on enhancing the visibility and diagnostic accuracy of root canal systems on periapical X-rays, with particular attention to combining digital image enhancement with clinician-based and technology-assisted interpretation. In doing so, we seek to contribute to improved diagnostic reliability, reduce the incidence of missed canals, and support better long-term outcomes in endodontic care.

A promising method for improving radiographic interpretation is Contrast-Limited Adaptive Histogram Equalization (CLAHE), which enhances local contrast by operating on small image regions while limiting noise over-amplification [18]. Unlike global Histogram Equalization (HE), CLAHE improves the visibility of fine structures such as root canal outlines and periapical bone patterns without introducing artifacts. It has been shown to increase both the PSNR and subjective visibility in dental X-rays [19], and to enhance canal morphology delineation in sclerosed or complex regions, serving as an effective preprocessing step for manual and AI-based analysis [20].

Due to its non-destructive, real-time capabilities, CLAHE is well suited for clinical settings requiring rapid image enhancement without the loss of diagnostic accuracy. When combined with clinician expertise or AI tools, it can elevate the diagnostic value of 2D radiographs to near-advanced imaging levels [21,22].

Building on this, we propose Total Variation–Contrast-Limited Adaptive Histogram Equalization (TV-CLAHE), which integrates CLAHE with Total Variation (TV) denoising to improve local contrast while reducing noise and preserving fine anatomical edges essential for accurate root canal visualization.

In this study, we evaluate and compare several image enhancement techniques, including the proposed TV-CLAHE method, for their ability to improve root canal visibility on periapical radiographs. The diagnostic performance of TV-CLAHE is benchmarked against both conventional and advanced enhancement algorithms, using CBCT as the reference standard.

## 2. Materials and Methods

### 2.1. Study Design and Ethical Approval

This study was designed as a retrospective observational analysis of dental radiographs. A total of 60 dental radiographic images of incisors (1 per patient) were retrospectively collected from the University Dental Clinic Department of Endodontics. Radiographs were performed during standard diagnostic procedures in patients referred for dental treatment. The images were postprocessed using six enhancement techniques: a global method (Histogram Equalization, HE), local adaptive methods (CLAHE and Global CLAHE), an optimization-based approach combining CLAHE with the Pelican Optimization Algorithm (CLAHE-POA), a gradient-based technique using fractional derivatives (K-CFDO), and a hybrid method proposed in this study (TV-CLAHE), which integrates CLAHE with Total Variation denoising to enhance contrast while suppressing noise. As a result, 420 images were obtained, including 60 original images and their enhanced versions. The resulting images were assessed by medical professionals.

All patients were adults (age range 18–71 years) with fully developed permanent teeth. The inclusion criteria for radiograph selection were as follows:

- Sufficient image quality for diagnostic evaluation (proper exposure, contrast, and no motion artifacts).
- Complete root formation (fully developed root canals with closed apices).
- No history or radiographic evidence of dental trauma to the tooth in question.
- No concomitant diseases or pathological lesions affecting the tooth or surrounding structures.
- Images where clinical detection of the number of canal roots was doubtful.
- Patient age between 18 and 71 years at the time of imaging.

Radiographs not meeting these criteria (e.g., poor image quality or teeth with open apices, trauma, or pathology or obvious roots visibility) were excluded from analysis. Prescreening was made by the team of doctors from the Department of Endodontics during standard diagnostic medical procedures. All images were deidentified and did not contain any personal patient information. This study received approval from the institutional review board and Local Bioethic committee permission number: 106/KBL/OIL/2024 from 12 December 2024 and due to its retrospective nature using careful deidentification radiographs, the requirement for informed consent was formally waived. The study was conducted in accordance with the Declaration of Helsinki and relevant institutional guidelines.

### 2.2. Image Acquisition and Viewing Conditions

All radiographs were intraoral periapical X-ray images obtained in the years 2022–2024 as part of routine dental care using standard digital radiography systems. The images were stored in DICOM format and were reviewed at their full original resolution without compression. For image display, we utilized high-resolution medical-grade monitors (Bracco, 4K ultra-high-definition, Bracco Imaging S.p.A., Milan, Italy) with a native resolution of 3840 × 2160 pixels. These 4K monitors are designed for diagnostic imaging and were calibrated to the DICOM Part 14 Grayscale Standard Display Function to ensure accurate and consistent luminance and contrast rendering across images. Each monitor’s brightness and contrast settings were standardized for radiographic viewing, and observers were allowed to use typical image processing tools (zoom, pan, and window/level adjustments) during evaluation.

#### Radiographic Imaging Protocol

Periapical radiographs were obtained using a dental X-ray system (Gendex 765 DC with RVG GXS-700, Gendex Dental System, Des Plaines, IL, USA 2016). Digital images were acquired at 65 kVp and 7 mA with a mean exposure time of 0.125 s, image dimensions of 1200 × 1600 pixels, and a pixel size of 0.018 mm. Digital images were saved in 16-bit digital imaging and communications in medicine (DICOM) format in the local PACS. Cone-beam computed tomography (CBCT) scans were acquired using the Orthopantomograph KaVo OP 3D Pro z Ceph 13*15 (Instrumentarium Dental, PaloDEx Grup Oy, Tuusula, Finland 2017). Scans were performed with the patient seated, using standard head positioning aids and bite block to ensure stability. The imaging protocol included a field of view (FOV) of either 5 × 5 cm or 8 × 15 cm, selected based on clinical indication. A voxel size of 125 µm was used for all standard scans. Exposure parameters were set to 90 kV, 6–8 mA, and a scan time of approximately 10 s, with a full 360° rotation of the gantry. Image acquisition and reconstruction were performed using the manufacturer’s software, and all scans were evaluated in multiplanar views (axial, sagittal, and coronal) on calibrated diagnostic monitors.

The radiographic assessments were performed in a dedicated radiology reading room under controlled ambient lighting. The room was dimly lit—not completely dark—to simulate standard diagnostic conditions. Ambient illumination was maintained at a low level (approximately 25–40 lux) throughout the reading sessions, in line with recommended radiology viewing environment standards. This low lighting level minimizes glare on the monitor and reduces observer eye strain while preventing complete darkness that could cause monitor luminance to appear dazzling. All observers adapted to the ambient light conditions for several minutes before starting the evaluation to ensure optimal visual acuity.

### 2.3. Image Enhancement Methods

Image enhancement procedures were implemented in Python 3.11.9 using the libraries OpenCV (version 4.11.0.86) and SciPy (version 1.15.2). The processing pipeline incorporated a series of enhancement methods, described below.

- Histogram Equalization (HE)—as a baseline method, global Histogram Equalization was applied to improve contrast distribution by redistributing intensity levels uniformly across the entire dynamic range. This was implemented using OpenCV’s cv2.equalizeHist () function.
- Contrast-Limited Adaptive Histogram Equalization (CLAHE)—applied using OpenCV’s cv2.createCLAHE () function with the following parameters: a clip limit of 2.0 and a tile grid size of 8 × 8. CLAHE improves local contrast while preventing over-amplification of noise through the clip limit.
- CLAHE with Pelican Optimization Algorithm (CLAHE-POA)—proposed by Haddadi et al. [23], it integrates CLAHE with the Pelican Optimization Algorithm (POA) to achieve data-driven parameter tuning. POA iteratively optimizes the CLAHE clip limit by minimizing a composite objective function incorporating PSNR, SSIM, and MSE. This adaptive approach ensures context-sensitive contrast enhancement while mitigating the risk of noise amplification and manual bias.
- K-Caputo Fractional Differential Operator (K-CFDO) approach—K-CFDO, developed by Aldoury et al. [24], applies a k-Caputo Fractional Differential Operator to perform the controlled enhancement of image gradients. Fractional-order derivatives are computed using k-symbol formulations combined with probabilistic weighting, enabling selective amplification of local variations. In this study, K-CFDO was included as an alternative method, representing advanced enhancement approaches unrelated to CLAHE-based techniques, and serving to broaden the comparative analysis of enhancement strategies.
- Global Contrast-Limited Adaptive Histogram Equalization (G-CLAHE)—developed by Namazi Nia and Shih [25], it employs an iterative local enhancement strategy constrained by global image characteristics. Local enhancement is guided by maximizing similarity between the Locally Enhanced Image (LEI) and its Globally Equalized Image (GEI) counterpart. The procedure terminates when further processing reduces this similarity, thereby preserving global contrast while refining local details.
- CLAHE with Total Variation denoising (TV-CLAHE)—proposed in this study, it combines multi-stage CLAHE processing with Total Variation (TV) denoising and spatial normalization to improve local contrast while controlling noise. The method consists of the following steps:
  1. Initial CLAHE enhancement is applied using a clip limit of 1.5 and a tile grid size of 8 × 8 to locally enhance contrast.
  2. Spatial intensity normalization is performed by dividing the image by its Gaussian-blurred version (σ = 50, minimum floor = 5), which suppresses large-scale intensity variations while retaining local structures.
  3. Total Variation denoising is then applied to the normalized image using the Chambolle algorithm (weight = 0.1). This step reduces noise and minor artifacts while preserving important edges.
  4. Final CLAHE enhancement is applied again with the same parameters to restore local contrast and improve perceptual visibility after denoising.

### 2.4. Observer Characteristics and Qualitative Image Evaluation Procedure

Four experienced observers independently evaluated the radiographs: two radiologists and two dental clinicians. The radiologist observers were board-certified radiologists with 15 and 6 years of experience in interpreting medical and dental imaging, respectively. The dental observers were licensed dentists (one endodontist and one general dentist) with 20 and 24 years of clinical experience, respectively, both having substantial familiarity with dental radiograph interpretation. Prior to the study, the evaluation criteria and definitions of the radiographic findings of interest were reviewed and agreed upon by all observers to ensure a common understanding of what the assessment goal is in each image (recognition of the dental canals). All observers were blinded to any clinical information about the patients (such as symptoms or dental history) to prevent bias; they based their assessments solely on the image content, and each observer was blinded to the others’ interpretations during the initial review phase.

The sequence of image review was randomized to eliminate any potential order effects. Each radiograph file was assigned a random identification code, and images were presented in a random order unique to each reading session. A total of 420 radiographs were evaluated, consisting of 60 original images and their enhanced versions generated by the previously described methods.

#### 2.4.1. The Evaluation of Images Proceeded as Follows

Four observers (two general dentists and two radiologists), blinded to the enhancement method, independently assessed the images. The evaluation was conducted individually without any consultation between observers. For each radiograph, observers recorded the number of visible root canals in accordance with the study criteria.

The visibility of root canals on conventional radiographs was assessed relative to the cone-beam computed tomography (CBCT) images, which served as the gold standard and were assumed to provide 100% visibility of root canals. Detection accuracy for each enhancement method was calculated as the percentage of root canals correctly identified on the radiographs compared to the total number of canals visible on the corresponding CBCT images.

#### 2.4.2. Diagnostic Performance Metrics Calculation

The assessment of root canal morphology was conducted by comparing observer evaluations with a reference standard established using cone-beam computed tomography (CBCT). CBCT imaging was considered the gold standard due to its superior ability to visualize root canal anatomy comprehensively and accurately. Each canal configuration observed on CBCT was considered 100% accurate and assigned a baseline score of 100 points, reflecting both the correct identification of the number of canals and the qualitative description of their shape and course.

The evaluators analyzed a total of 420 periapical radiographs processed with different enhancement techniques and described the number and morphology of root canals. If the observer’s description differed from the CBCT reference in terms of canal count or morphological classification (e.g., describing the canal as blunt, bulbous, tapered, or irregular when not consistent with the CBCT), a penalty of 10 points was applied for each incorrect parameter.

Each image was independently evaluated by four observers. The final score per case was calculated as the average of the individual scores given by the four evaluators. These scores served as the basis for statistical comparisons between enhancement methods.

### 2.5. Subjective Image Quality Assessment Using Own Scale

To assess the subjective visibility of root canals across enhanced radiographic images, we developed an own scale inspired by a 5-point Likert scale on the basis of the evaluative framework outlined by Precht et al. [26], which emphasizes structured observer feedback in image enhancement studies. In contrast to traditional bipolar Likert formats, we implemented a symmetric, positive-only scale to focus on the degree of improvement in root canal visibility relative to the original image. The evaluators were instructed to rate each enhanced image using the following criteria: 1 = Same, 2 = Slightly better, 3 = Much better, 4 = Very good, 5 = Excellent. This approach provides a quantifiable, reproducible method for visual quality assessment, aligned with human observer-based evaluation strategies used in prior radiological image studies.

Own scale inspired by Likert scale for root canal visibility evaluation

1. Same: No perceptible improvement in the visibility of root canals compared to the original image.
2. Slightly better: Minor enhancement in root canal visibility.
3. Much better: Noticeable improvement; canal structures are clearer and easier to interpret.
4. Very good: Significant enhancement in visibility, allowing confident assessment of canal anatomy.
5. Excellent: Optimal visibility of root canals, surpassing original image quality and providing diagnostic clarity.

Observer ratings were summarized using descriptive statistics. Median ratings were calculated per observer and method to reflect individual scoring tendencies, while overall medians across all ratings were computed to assess global method performance.

Due to the ordinal nature of the data, non-parametric tests were employed. Inter-observer reliability was assessed using the Intraclass Correlation Coefficient (ICC), model ICC (2,k), which measures absolute agreement among raters.

Differences between enhancement methods were evaluated using the Friedman test, appropriate for repeated measures and ordinal data. Statistical significance was defined as *p* < 0.05.

When significant overall differences were detected, Wilcoxon signed-rank tests with Bonferroni correction were applied for post hoc pairwise comparisons to identify specific method pairs showing significant differences.

### 2.6. Quantitative Evaluation

Quantitative evaluation was performed to provide an objective and reproducible comparison of the enhancement methods, complementing the clinical (qualitative) assessment performed by human observers. This approach allowed for the analysis of information content, and local enhancement consistency using standardized image quality metrics.

To assess the impact of enhancement, the following metrics were computed across the entire image:

- Peak Signal-to-Noise Ratio (PSNR), defined as follows:
  $$\mathrm{PSNR}=10\cdot \log_{10}\left(\frac{{\mathrm{MAX}}^{2}}{\mathrm{MSE}}\right)$$
  where MAX represents the maximum possible pixel intensity, and MSE is the mean squared error between the original and enhanced images. Higher PSNR values indicate greater similarity and lower distortion.

- Signal-to-Noise Ratio (SNR), calculated as follows:
  $$\mathrm{SNR}\;=10\cdot \log_{10}\left(\frac{\mu^{2}}{\sigma^{2}}\right)$$
  where μ is the mean pixel intensity and σ is the standard deviation of the image. Higher SNR values reflect lower relative noise levels and thus better global image clarity.

- Shannon entropy, computed as follows:
  $$H=-\sum_{i=0}^{255}p\left(i\right)\cdot \log_{2}\left[p\left(i\right)\right]$$
  where *p*(*i*) represents the normalized histogram (probability) of each pixel intensity. Higher entropy indicates greater information content and contrast diversity.

- Rényi entropy, with order α = 2, defined as follows:
  $$H_{\alpha }=\frac{1}{1-\alpha }\log_{2}\left(\sum_{i=0}^{255}p{\left(i\right)}^{\alpha }\right)$$
  which provides an alternative measure emphasizing dominant intensity values and contrast structures.

- Structural Similarity Index (SSIM), expressed as follows:
  $$\mathrm{SSIM}\left(x,y\right)=\frac{\left(2\mu_{x}\mu_{y}+C_{1}\right)\left(2\sigma_{xy}+C_{2}\right)}{\left(\mu_{x}^{2}+\mu_{y}^{2}+C_{1}\right)\left(\sigma_{x}^{2}+\sigma_{y}^{2}+C_{2}\right)}$$
  where μ*_x_* and μ*_y_* are the mean intensities, σ*_x_* and σ*_y_* the variances, and σ*_xy_* the covariance of the original and enhanced images *x* and *y*, respectively. *C*_1_ and *C*_2_ are stabilizing constants. SSIM evaluates perceptual similarity by jointly considering luminance, contrast, and structural fidelity.

### 2.7. Statistical Analysis

Prior to hypothesis testing, the distributions of metric values were assessed for normality using the Shapiro–Wilk test. As the majority of the distributions deviated from normality, non-parametric tests were selected for subsequent analysis.

Pairwise comparisons between enhanced images and the original reference were performed using the Wilcoxon signed-rank test, which is appropriate for paired and non-normally distributed data. For each computed metric (PSNR, SNR, Shannon entropy, Rényi entropy, and SSIM), tests were conducted between the original images and each enhancement method.

To account for multiple comparisons across methods and metrics, *p*-values were adjusted using the Bonferroni correction. Corrected *p*-values below the significance threshold (α = 0.05) were considered statistically significant. Additionally, descriptive statistics, including the mean and standard deviation (SD), were computed for each metric across all images. All statistical analyses were performed in Python (version 3.11.9) using the SciPy (version 1.15.2) and Statsmodels (version 0.14.4) libraries.

## 3. Results

### 3.1. Qualitative Evaluation

In the evaluation of the original (unprocessed) CR images, the root canal detection rates were relatively low, ranging between 51 and 56% among the four evaluators.

The application of enhancement algorithms resulted in measurable improvements in the detection accuracy:

- CLAHE increased the detection rates modestly to 58–64%.
- CLAHE-POA showed a moderate increase, ranging from 59 to 64%.
- G-CLAHE achieved a major breakthrough, boosting the detection rates up to 90–92%, approaching CBCT-level visibility.
- HE resulted in small improvements to around 57–61%.
- KCFDO did not improve performance compared to the original, with accuracies similar to baseline (around 49–53%).
- TV-CLAHE achieved the highest performance, with detection rates ranging between 93 and 98%.

From a clinical standpoint, it is important to note that only complex cases identified during prescreening were included in the study—those characterized by unusual anatomical shapes, intricate morphology, or suboptimal imaging conditions due to overlapping structures. The details of canal configurations and X-ray features are provided in Table 1.

Cone-beam computed tomography (CBCT) served as the reference modality, representing 100% visibility of root canals. Overall, the TV-CLAHE method nearly matched the CBCT diagnostic accuracy while still using conventional 2D radiographic data. G-CLAHE also provided substantial improvements, consistently exceeding 90% detection accuracy across observers.

As shown in Table 2, the reference baseline detection accuracy for dental canals on the original radiographs was approximately 50% (slightly, consistent with previous reports) [27].

The application of advanced image enhancement techniques significantly improved the detection rates. Among the standard methods, Contrast-Limited Adaptive Histogram Equalization (CLAHE) and its derivatives (CLAHE-POA and G-CLAHE) demonstrated notable improvements, with G-CLAHE achieving detection rates exceeding 90% across all evaluators. Traditional Histogram Equalization (HE) provided only moderate improvement. KCFDO did not enhance the detection performance, maintaining results close to the baseline.

Our proposed methods (TV-CLAHE) further increased the detection accuracy, achieving the highest rates, reaching up to 98% for radiologists. These results highlight the critical role of advanced image processing in enhancing dental structure visibility on radiographs.

Median ratings indicated clear differences in perceived enhancement quality across methods, as shown in Table 3. It presents the median ratings of root canal visibility enhancement methods as evaluated by four observers (Dentist 1, Dentist 2, Radiologist 1, and Radiologist 2) using a symmetric scale ranging from 1 (Same) to 5 (Excellent). Each cell reflects the median score assigned by each evaluator, with the “Global Median” representing the overall median across all ratings for each method. TV-CLAHE received the highest global median score (5.0), followed by G-CLAHE (4.0), suggesting superior visibility improvements. Moderate median scores were observed for CLAHE, CLAHE-POA, and HE (3.0), while KCFDO received the lowest global median (2.0), indicating limited perceived enhancement.

Inter-observer reliability, assessed using ICC (2,k), yielded a value of 0.650 (95% CI: 0.59–0.71, *p* < 0.001), reflecting moderate agreement among raters.

The Friedman test revealed statistically significant differences between methods (*χ*^2^ = 211.94, *p* < 0.001). Post hoc analysis using Wilcoxon signed-rank tests with Bonferroni correction identified significant differences between most method pairs. No significant differences were observed among CLAHE, CLAHE-POA, and HE, suggesting comparable subjective performance.

**Figure 1:**
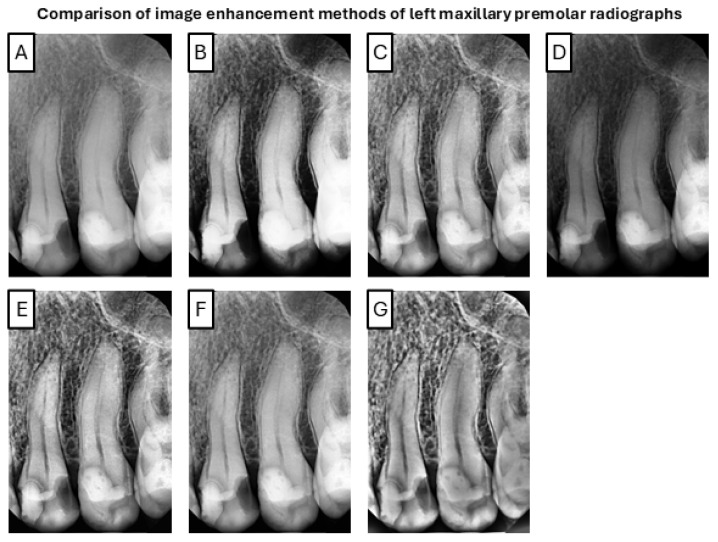
Representative example of premolar radiographs processed using the evaluated enhancement methods. Enhanced images demonstrate varying levels of contrast improvement and anatomical detail visibility. (**A**) Original, (**B**) HE, (**C**) CLAHE, (**D**) KFCDO, (**E**) G-CLAHE, (**F**) CLAHE-POA, (**G**) TV-CLAHE.

For qualitative assessment, a representative example of the original and enhanced radiographs was selected and is presented in Figure 1. The comparison includes images processed using all the enhancement methods described in this study. Notably, substantial visual differences were observed across the methods. The original image exhibited limited contrast and poor visibility of fine anatomical structures, including the root canals.

**Figure 2:**
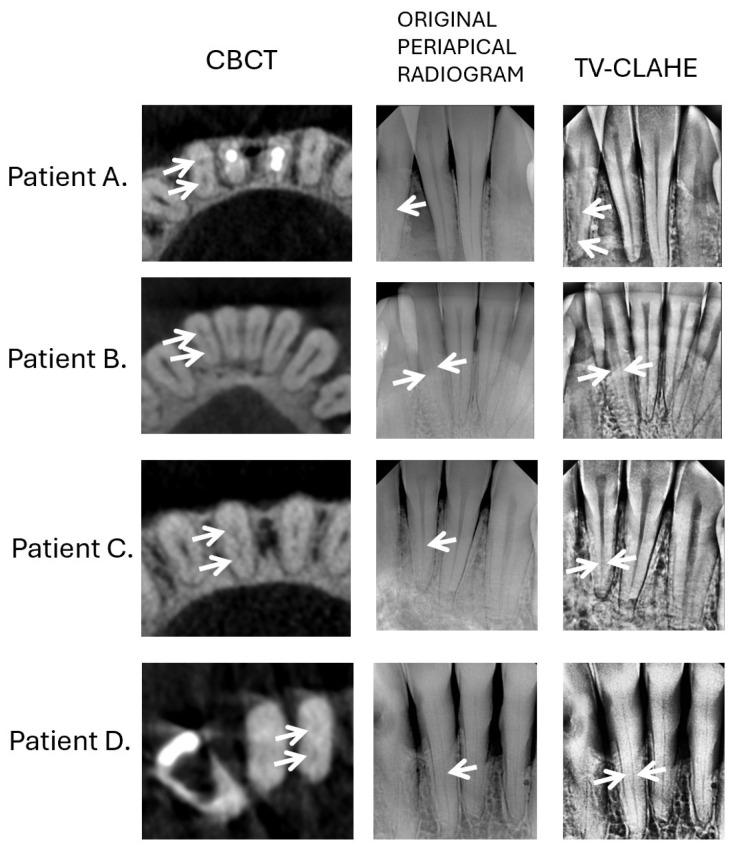
Comparison of root canal visualization in four patients (Patient A–D). Left column: axial CBCT images; middle column: original periapical radiographs; right column: images enhanced using the proposed TV-CLAHE method. All examples show mandibular lower incisors in the axial (CBCT) and coronal (periapical) planes. Arrows indicate root canals, with enhanced visibility in the processed images.

In addition, Figure 2 presents a comparative visualization of root canals in four different patients, highlighting the effectiveness of the proposed enhancement. The figure contrasts axial CBCT images, original periapical radiographs, and TV-CLAHE-enhanced images, clearly demonstrating improved canal visibility in the processed outputs.

### 3.2. Quantitative Results

The quantitative evaluation results for each enhancement method are summarized in Table 4. Metrics included PSNR, SNR, Shannon entropy, Rényi entropy, and SSIM, expressed as mean and standard deviation across all evaluated images. Statistically significant differences compared to the original images, determined after Bonferroni correction, are marked with “*”.

CLAHE-POA achieved the highest PSNR, followed by CLAHE and G-CLAHE. These methods preserved pixel-level similarity most effectively. The SNR values remained generally stable across most methods. However, HE and KCFDO had a significantly lower SNR compared to the original images, while TV-CLAHE recorded the highest SNR, indicating improved signal clarity and noise suppression. Entropy analysis showed varied results across the methods. The CLAHE-based methods and TV-CLAHE increased the Shannon and Rényi entropy, suggesting enhanced local contrast and greater intensity diversity. In contrast, HE and KCFDO resulted in decreased entropy compared to the original images, reflecting reduced intensity variation. SSIM was highest for CLAHE-POA, indicating the best preservation of structural similarity relative to the original images. The lower SSIM values observed for G-CLAHE and TV-CLAHE reflect their emphasis on local contrast enhancement over global structural fidelity.

## 4. Discussion

In the present study, we evaluated the effects of various image enhancement techniques on the detection accuracy of dental root canals in conventional radiographic (CR) images. The performance differences observed can be explained based on established image processing principles and the findings from both dental imaging and broader computer vision research.

### 4.1. HE

Standard HE increased the detection rates only modestly (from 51–56% to approximately 57–61%). HE is a global contrast enhancement technique that redistributes pixel intensity values across the entire dynamic range of the image [28]. Although this method improves the overall contrast, it tends to amplify noise, especially in low-signal regions, and can lead to over-saturation of brighter areas. Quantitative analysis confirmed that HE produced relatively low PSNR and SSIM values compared to other methods, indicating reduced pixel-wise similarity and poorer structural preservation. In dental radiographs, such global enhancement may obscure fine anatomical details, including small root canals, due to increased background noise [19,29]. Consequently, HE provided only a limited improvement in root canal visibility.

### 4.2. CLAHE

CLAHE, a localized variant of Histogram Equalization [30], showed a better but still moderate improvement (58–64%). CLAHE enhances contrast within small tiles of the image while preventing over-amplification through histogram clipping. This localized adjustment helps to preserve subtle structures, such as root canals, without overwhelming the image with noise. Pisano et al. [29] demonstrated that CLAHE enhances fine detail visibility in low-contrast medical images. However, some noise amplification at tile borders may still occur, explaining why the gain in detection remained moderate. Overall, CLAHE improved the local contrast and visibility compared to HE, but its effectiveness remained moderate relative to G-CLAHE and TV-CLAHE.

### 4.3. CLAHE-POA

The CLAHE-POA method, employing the Pelican Optimization Algorithm to tune the CLAHE parameters [23], achieved a moderate improvement (59–64%). The optimization process produced more balanced contrast enhancement compared to standard CLAHE, reflected in slightly higher PSNR and entropy values. However, as the optimization was not specifically guided by root canal visibility but rather by general image quality criteria, the improvement in detection performance remained modest. This highlights the limitation of optimization when the objective function does not precisely align with the diagnostic task.

### 4.4. G-CLAHE

G-CLAHE achieved a substantial breakthrough in performance, raising the detection rates to 90–92%. The quantitative results showed that G-CLAHE achieved the highest Shannon entropy, reflecting improved intensity diversity and local detail visibility. This method integrates global and local Histogram Equalization to simultaneously preserve the overall brightness consistency and local contrast enhancement [25]. Unlike pure CLAHE, which can cause brightness inconsistencies across the image, G-CLAHE maintains a stable global appearance while emphasizing local features. In dental imaging, maintaining both global and local features is critical, as the brightness of root canals relative to the tooth and bone background plays an important role in detectability [25]. This dual enhancement approach likely explains why G-CLAHE allowed near-CBCT-level visibility.

### 4.5. KCFDO

K-CFDO did not improve the detection rates, which remained at 49–53%. This method, based on the application of the k-Caputo Fractional Differential Operator [24], aims to enhance image quality by adjusting pixel intensity distributions through fractional calculus. However, quantitative analysis indicated lower PSNR, SNR, and entropy values compared to other enhancement methods. These findings suggest that K-CFDO may have introduced excessive smoothing or amplified non-relevant textures, which could obscure fine anatomical structures such as root canals. This result highlights that general-purpose enhancement techniques may not directly translate to improved diagnostic performance in tasks requiring subtle detail recognition.

### 4.6. TV-CLAHE

TV-CLAHE achieved the highest detection rates, ranging from 93% to 98%. The method integrates multi-stage CLAHE processing TV denoising and spatial normalization to enhance local contrast while reducing noise. TV denoising was implemented using the Chambolle algorithm [31], which minimizes Total Variation by solving an optimization problem that reduces minor fluctuations in pixel intensity while preserving significant edges.

The quantitative results confirmed the effectiveness of this approach. TV-CLAHE recorded the highest SNR (2.60) and Rényi entropy (7.30), indicating improved signal clarity and contrast. Shannon entropy was also among the highest (7.54), reflecting enhanced intensity diversity. Although the PSNR and SSIM were lower compared to the CLAHE-based methods, this is consistent with the more aggressive local enhancement and structural adjustment required to reveal subtle features. Such strategies are increasingly advocated in dental imaging to maximize diagnostic utility while minimizing artifacts [19,25].

In this study, we demonstrated that the application of CLAHE and its advanced variants to periapical radiographs significantly enhances the visualization of root canal systems. Our results show that, following enhancement with CLAHE-based methods, experienced clinicians were able to detect root canals with 96% accuracy, confirmed against CBCT as the reference standard. These findings support CLAHE-based techniques as a robust and low-cost image enhancement tool, capable of significantly augmenting radiographic interpretation in endodontic diagnostics.

The utility of CLAHE and related methods in dental imaging has been previously established. Rahmi-Fajrin et al. evaluated several image enhancement techniques, including CLAHE, for periapical radiographs [18]. Their study found that CLAHE significantly increased the PSNR and reduced the MSE, resulting in sharper, more diagnostically useful images [18]. These findings mirror our own, suggesting that local contrast amplification approaches, such as those used in CLAHE-based methods, are particularly effective for making root canal systems more distinguishable against surrounding bone and dentin.

Our results are also in line with more advanced morphological image enhancement techniques. Mello Román et al. proposed a multiscale top-hat transformation with geodesic reconstruction (MSTHGR) to enhance panoramic dental radiographs, reporting superior edge clarity and contrast improvement compared to CLAHE and other global equalization methods [21]. While CLAHE-based approaches remain widely accessible and easy to implement, multiscale morphological techniques like MSTHGR suggest a valuable direction for future enhancement pipelines aimed at even finer anatomical delineation in 2D dental imaging.

Beyond enhancement, our findings relate closely to recent work in automated root canal detection using artificial intelligence. Zhang et al. developed a lightweight convolutional neural network (CNN) incorporating receptive field blocks for detecting C-shaped root canals on panoramic radiographs, achieving an accuracy of 98.4% [32]. Their study underscores that not only is AI capable of detecting complex canal morphologies, but also that image enhancement is often a prerequisite for maximizing model performance. It is noteworthy that in our study, a similar level of accuracy was achieved through expert interpretation when CLAHE-based enhancement was applied—indicating that human diagnosis, when properly supported by enhancement, can reach the diagnostic quality typically associated with CNN models. Similarly, Shetty et al. evaluated multiple machine learning (ML) models for detecting MB2 canal orifices in axial CBCT images, reporting high performance with neural networks (AUC 0.896) and support vector machines (precision 86.8%, recall 92.5%) [33].

In line with this, Ari et al. employed a U-Net-based deep learning framework for the automatic segmentation of root canal fillings, caries, and other features in periapical radiographs, achieving an F1-score of 0.98 for detecting root canal fillings [22]. This shows that not only the detection, but also the precise segmentation of canal-related structures is feasible in 2D radiographs, especially when preprocessing such as CLAHE-based methods is employed. Our results suggest that radiologist performance in detecting root canals may benefit equally from CLAHE-driven preprocessing, without necessarily requiring full automation.

Further supporting this, Jin et al. introduced a deep learning model based on YOLOv5 to evaluate the quality of root canal obturations on periapical X-rays [20]. The system not only detected canal fill terminations but also judged their adequacy with over 92% accuracy for correctly filled canals [20]. The system’s strength lay in its ability to localize the apical end of the canal—an anatomical feature that becomes far more discernible when enhancement techniques like CLAHE-based processing are applied. The consistency of our clinician-based findings with those achieved using AI detection reinforces the clinical viability of CLAHE-derived methods in real-world diagnostics.

Interestingly, not all improvements in canal visualization rely on software techniques. Govind et al. investigated a chitosan-based endo-radiopaque contrast solution (CERS) to physically enhance canal visualization during radiography [34]. Their solution penetrated deeper into root canals and improved the visibility of canal anatomy on radiographs when compared to conventional agents [34]. This physical approach to enhancement further validates the principle that clarifying canal structures—whether digitally or chemically—yields better diagnostic outcomes. Our work offers a software-based parallel to this principle, demonstrating that even without physical agents, digital enhancement via CLAHE-based enhancement can deliver near-parity in diagnostic accuracy with CBCT.

Taken together, these findings point to several important implications:

1. CLAHE-based methods are clinically valuable tools for enhancing the diagnostic quality of periapical radiographs in root canal evaluation. Our 96% detection accuracy demonstrates its effectiveness in aiding clinicians to visualize canal anatomy otherwise difficult to interpret.
2. The performance of CLAHE-enhanced radiographs and advanced variants is comparable to or supportive of AI-based diagnostic systems, suggesting it could serve as a preprocessing standard for CNN pipelines or hybrid AI–clinician workflows.
3. When compared to advanced enhancement techniques (e.g., MSTHGR, RFB-CNNs), CLAHE-based methods offer a practical, low-barrier alternative that still delivers significant clinical benefit, especially in settings where AI or contrast agents are unavailable.

### 4.7. Limitations and Future Directions

This study has several limitations. The quantitative analysis relied on a limited set of metrics, such as the PSNR, SNR, entropy, and SSIM. Although useful for assessing general image quality, these indicators do not fully reflect clinical perceptibility or the subtle anatomical details critical for root canal detection. For example, the SNR—defined as the ratio of mean signal intensity within the imaged object to the standard deviation of background noise—is widely used in medical imaging but has notable limitations. For instance, it is not always feasible to assess noise in images that consist solely of anatomical structures such as organs and tissues. Additionally, the SNR is influenced by the absorption of X-ray radiation by the tissues, which affects image brightness. However, a lower SNR—or reduced brightness—does not necessarily imply a loss in image quality, particularly when quality is defined as the ability to discern fine details or extract meaningful diagnostic information. Moreover, medical image readers often operate in varying environments (e.g., different lighting conditions, viewing angles, and distances), where the impact of tissue-specific image degradation and unmeasured distortions can further complicate quality assessment.

To address these limitations, future work should explore more targeted and objective evaluation criteria, including anatomical detection metrics and automated analysis methods. In addition, combining CLAHE-based enhancement with texture-based analysis techniques, such as fractal dimension or radiomic signatures, may provide a more comprehensive assessment of image content. Texture analysis has long been a proven tool for evaluating various medical images, including dental radiographs [35]. Texture reflects the structural organization of the visualized tissues, and texture parameters enable quantitative and objective analysis of images, including their quality assessment [36]. The use of texture-based metrics for assessing radiograph quality could address some limitations of conventional measures such as the SNR or PSNR, which primarily depend on signal brightness, by also capturing the structural and morphological features of the imaged tissues. Integrating enhanced images as preprocessing inputs for CNN-based detection models also presents a promising direction. Such hybrid approaches could leverage the strengths of both human interpretation and artificial intelligence, ultimately supporting the development of assistive systems that offer both rapid automated analysis and the contextual judgment of clinicians. Future studies should incorporate targeted anatomical detection metrics, such as canal visibility scoring, identification rates of specific configurations (e.g., Vertucci Types II–IV), and sensitivity/specificity in detecting accessory canals compared to CBCT. Additionally, using annotated benchmark datasets and observer consistency measures (e.g., Cohen’s κ) may improve reproducibility. These task-specific criteria could offer a more direct assessment of diagnostic performance than general image quality metrics alone.

## 5. Conclusions

In this study, we systematically evaluated a range of image enhancement techniques for improving root canal visibility in periapical radiographs, including global, local, and advanced CLAHE-based methods. While standard Histogram Equalization provided only limited benefits, localized enhancement with CLAHE significantly improved the detection rates. Further refinement through advanced variants, notably G-CLAHE and TV-CLAHE, resulted in near-CBCT-level visibility, with TV-CLAHE achieving the highest detection rates (93–98%). Quantitative analysis confirmed that these CLAHE-based methods offered superior performance in enhancing local contrast and suppressing noise while maintaining anatomical detail.

In summary, enhancement methods that combine localized contrast adjustment, edge preservation, and noise suppression produced the most substantial improvements in root canal detection. Specifically, methods such as G-CLAHE and TV-CLAHE enhanced the visibility of fine anatomical structures while minimizing the amplification of irrelevant noise. This aligns with established principles in image processing, where balancing contrast, edge preservation, and noise control is critical for diagnostic quality. In the context of dental radiography, where images often exhibit low contrast and fine linear structures, careful algorithmic design targeting these unique features remains essential for effective enhancement.

## Figures and Tables

**Table 1:** Canal configuration and X-ray characteristics of study teeth.

Vertucci Type	Configuration	Description	(*n* = 60)	Percentage (%)
Type I	1-1	One canal from chamber to apex	42	70%
Type II	2-1	Two canals merge into one	9	15%
Type III	1-2-1	Canal splits and rejoins	6	10%
Type IV	2-2	Two separate canals to two foramina	3	5%
Total	–	–	60	100%

**Table 2:** Diagnostic success rates (%) of different image enhancement methods across four evaluators (two general dentists and two radiologists). Each value represents the percentage of correct visual assessments compared to the CBCT reference standard in a binary classification task involving 420 cases. TV-CLAHE and G-CLAHE showed the highest overall success rates across all observers.

Enhancement Method	Dentist 1	Dentist 2	Radiologist 1	Radiologist 2
Original CR	61%	58%	53%	56%
CLAHE	63%	62%	58%	64%
CLAHE-POA	62%	64%	59%	63%
G-CLAHE	90%	92%	87%	92%
HE	67%	64%	57%	60%
KCFDO	50%	49%	53%	52%
TV-CLAHE	97%	95%	98%	93%

CR—conventional radiograph; CLAHE—Contrast-Limited Adaptive Histogram Equalization; CLAHE-POA—CLAHE with Pelican Optimization Algorithm; G-CLAHE—Global CLAHE; HE—Histogram Equalization; KCFDO—k-Caputo Fractional Differential Operator; TV-CLAHE—Total Variation–CLAHE.

**Table 3:** Median ratings of root canal visibility methods by four observers. ”Global Median” shows overall scores.

Enhancement Method	Dentist 1	Dentist 2	Radiologist 1	Radiologist 2	Global Median
CLAHE	3.0	3.0	2.0	3.0	3.0
CLAHE-POA	3.0	3.0	3.0	3.0	3.0
G-CLAHE	4.5	4.0	4.0	4.0	4.0
HE	3.0	3.0	2.0	3.0	3.0
KCFDO	1.5	2.0	2.0	1.0	2.0
TV-CLAHE	5.0	4.0	5.0	4.0	5.0

**Table 4:** Quantitative results for each enhancement method. Data are presented as mean (standard deviation). Asterisks indicate statistically significant differences (*p* < 0.05, Bonferroni corrected) compared to the original images.

Enhancement Method	PSNR	SNR	Shannon Entropy	Rényi Entropy	SSIM
Original	-	2.21 (0.47)	7.34 (0.23)	6.73 (0.45)	1.00 (0.00)
CLAHE	20.83 (1.54)	2.22 (0.37)	7.52 (0.15) *	7.05 (0.43) *	0.82 (0.03) *
CLAHE-POA	24.74 (2.19)	2.27 (0.43)	7.42 (0.18)*	6.86 (0.45) *	0.91 (0.03) *
G-CLAHE	18.46 (1.49)	2.15 (0.31)	7.61 (0.12) *	7.19 (0.43) *	0.73 (0.04) *
HE	17.89 (2.43)	1.57 (0.07) *	7.11 (0.25) *	6.66 (0.43) *	0.82 (0.08) *
KCFDO	14.85 (1.09)	1.50 (0.33) *	6.97 (0.27) *	6.22 (0.66) *	0.79 (0.07) *
TV-CLAHE	14.22 (1.44)	2.60 (0.24) *	7.54 (0.11) *	7.30 (0.16) *	0.72 (0.05) *

Abbreviations: PSNR = Peak Signal-to-Noise Ratio; SNR = Signal-to-Noise Ratio; SSIM = Structural Similarity Index Measure; *—statistically significant differences (*p* < 0.05).

## Data Availability

The original contributions presented in the study are included in the article, further inquiries can be directed to the corresponding authors.

## Abbreviations

The following abbreviations are used in this manuscript:

AI	Artificial intelligence
CBCT	Cone-beam computed tomography
ClAHE	Contrast-Limited Adaptive Histogram Equalization
CLAHE-POA	Contrast-Limited Adaptive Histogram Equalization with Pelican Optimization Algorithm
CERS	Chitosan-based endo-radiopaque solution
CR	Conventional radiograph
DOM	Dental operating microscope
DICOM	Digital imaging and communications in medicine
FOV	Field of view
G-CLAHE	Global Contrast-Limited Adaptive Histogram Equalization
HE	Histogram Equalization
ICC	Intraclass Correlation Coefficient
K-CFDO/KCFDO	k-Caputo Fractional Differential Operator
LEI	Locally Enhanced Image
MB2	Second mesiobuccal canal
MSE	Mean squared error
PACS	Picture Archiving and Communication System
POA	Pelican Optimization Algorithm
PSNR	Peak Signal-to-Noise Ratio
RFB	Receptive field block
SD	Standard deviation
SNR	Signal-to-Noise Ratio
SSIM	Structural Similarity Index
TV	Total Variation
TV-CLAHE	Total Variation–Contrast-Limited Adaptive Histogram Equalization
YOLOv5	You Only Look Once version 5 (a deep learning object detection model)
